# Genetic and environmental factors in Alzheimer’s and Parkinson’s diseases and promising therapeutic intervention via fecal microbiota transplantation

**DOI:** 10.1038/s41531-021-00213-7

**Published:** 2021-08-11

**Authors:** Hui Wang, Feng Yang, Shidong Zhang, Ruihua Xin, Yan Sun

**Affiliations:** 1 Department of Toxicology, School of Public Health, Lanzhou University, Lanzhou, Gansu China; 2 Lanzhou Institute of Husbandry and Pharmaceutical Sciences of Chinese Academy of Agricultural Sciences, Lanzhou, Gansu China

**Keywords:** Risk factors, Cellular neuroscience

## Abstract

Neurodegenerative diseases are characterized by neuronal impairment and loss of function, and with the major shared histopathological hallmarks of misfolding and aggregation of specific proteins inside or outside cells. Some genetic and environmental factors contribute to the promotion of the development and progression of neurodegenerative diseases. Currently, there are no effective treatments for neurodegenerative diseases. It has been revealed that bidirectional communication exists between the brain and the gut. The gut microbiota is a changeable and experience-dependent ecosystem and can be modified by genetic and environmental factors. The gut microbiota provides potential therapeutic targets that can be regulated as new interventions for neurodegenerative diseases. In this review, we discuss genetic and environmental risk factors for neurodegenerative diseases, summarize the communication among the components of the microbiota-gut-brain axis, and discuss the treatment strategy of fecal microbiota transplantation (FMT). FMT is a promising treatment for neurodegenerative diseases, and restoration of the gut microbiota to a premorbid state is a novel goal for prevention and treatment strategies.

## Introduction

Neurodegenerative diseases are characterized by neuronal impairment and loss of function that lead to the progressive impairment of cognitive function^1^. The misfolding and aggregation of specific proteins inside or outside cells are the major shared histopathological hallmarks of neurodegenerative diseases^2^; examples include misfolded α-synuclein deposits in Parkinson’s disease (PD), amyloid-β (Aβ) aggregates, and neurofibrillary tangles are formed from hyperphosphorylated tau in Alzheimer’s disease (AD)^3^, mutated huntingtin (HTT) in Huntington disease^4^, and TAR DNA-binding protein 43 (TDP-43) in amyotrophic lateral sclerosis (ALS)^5^. AD and PD are the two most common neurodegenerative disorders.

## The two most common neurodegenerative disorders: AD and PD

**Fig. 1:**
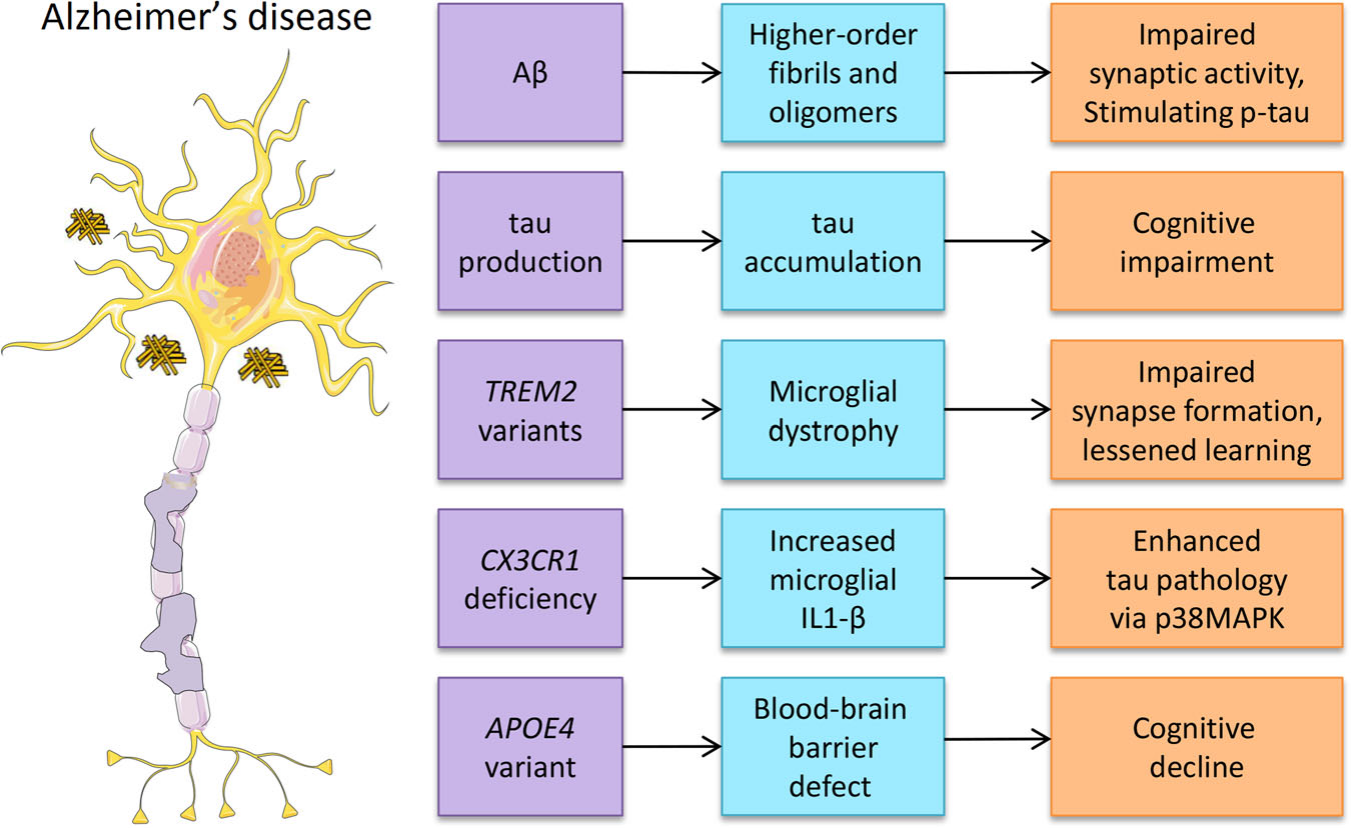
Associated markers or altered gene expression of AD. Aβ, tau, *TREM2* variants, targeted deletion of *CX3CR1*, and *APOE4* variants have all been associated with AD (left column). The middle and right columns show the downstream effects.

AD is characterized by cognitive dysfunction and progressive memory decline, and is caused by a complex interaction between genetic, lifestyle, environmental, and epigenetic factors^3^. The increased life expectancy worldwide has resulted in a significant increase in age-related diseases. Neurodegenerative disorders and dementia are increasing progressively with an incidence of 17.2 million people worldwide^1^. AD is one of the fastest-growing age-related diseases today^6^. Worldwide, 10% of people over the age of 65 years are affected by AD^7^, and after the age of 65, the risk of developing AD doubles every 5 years^8^. In the United States, 40% of people over 85 years old are cognitively impaired^9^; AD pathology probably contributes to 75–80% of these cases^10^, and more than 5 million individuals have AD^11^. The primary neuropathological criteria for AD diagnosis are the intracellular accumulation of hyperphosphorylated tau as neurofibrillary tangles and the extracellular deposition of Aβ as neuritic plaques^12^. Aβ is derived from Aβ precursor protein (APP) predominantly in endosomes by β-secretase and γ-secretase^13^. The autosomal dominant form of early-onset AD has been attributed to the overproduction of Aβ as the result of APP and presenilin 1 and 2 (PSEN1/2) mutations^14^. Synaptic activity both presynaptically and postsynaptically modulates neuronal Aβ release^15^. Aβ can aggregate into higher-order fibrils and oligomers, impair synaptic activity and cerebral capillary blood flow, and directly stimulate tau hyperphosphorylation^12^. Aβ accumulation may be a critical pathological process for the initiation of tau accumulation and neuroinflammation, which are the downstream events that may be the main drivers of neurodegeneration^12^. Tau protein is primarily expressed by brain neurons and is encoded by the *MAPT* gene^12^. The production of tau is related to the presence of amyloid proteins^16^, and the progression of tau pathology in AD requires Aβ deposition^17^. Tau spreads from cell to cell through neuronal connections, and this process can be facilitated by Aβ in animal models^17^. Tau pathology generally does not progress in the absence of amyloid pathology^18^. Elevating the Aβ level alone is sufficient to drive tau pathology in human neurons^19^. Recent research has suggested that tau spreads through neuronal communication pathways even in normal aging, and its spread is accelerated by the presence of Aβ in the human brain^20^. The rate of amyloid accumulation predicts the beginning of tau accumulation, whereas the rate of tau accumulation predicts the beginning of cognitive impairment^21^. Different individuals with “typical” AD may have distinct biochemical features of tau, including hyperphosphorylated soluble, oligomeric, seed-competent tau^22^. And microglial dysfunction contributes to the pathology of AD^23^ (Fig. 1). Microglia clear Aβ plaques and are involved in the development and progression of AD. When genes linked to AD risk (including *SPI1*, *CR1*, *TREM2*, *MS4As*, *ABCA7*, *CD33*, and *INPP5D*) are expressed in microglia, their phagocytic function is disrupted, and Aβ accumulates and activates the cascade that promotes subsequent neuronal degeneration^23,24^.

PD is the second most common neurodegenerative disease^25^. The incidence of PD increases progressively with age and affects 1 to 4% of individuals over the age of 60 years^11,26^ and over 5 million people worldwide^27^. AD and PD begin decades before the clinical manifestation of the first symptoms due to the formation of pathogenic protein aggregates.

**Fig. 2:**
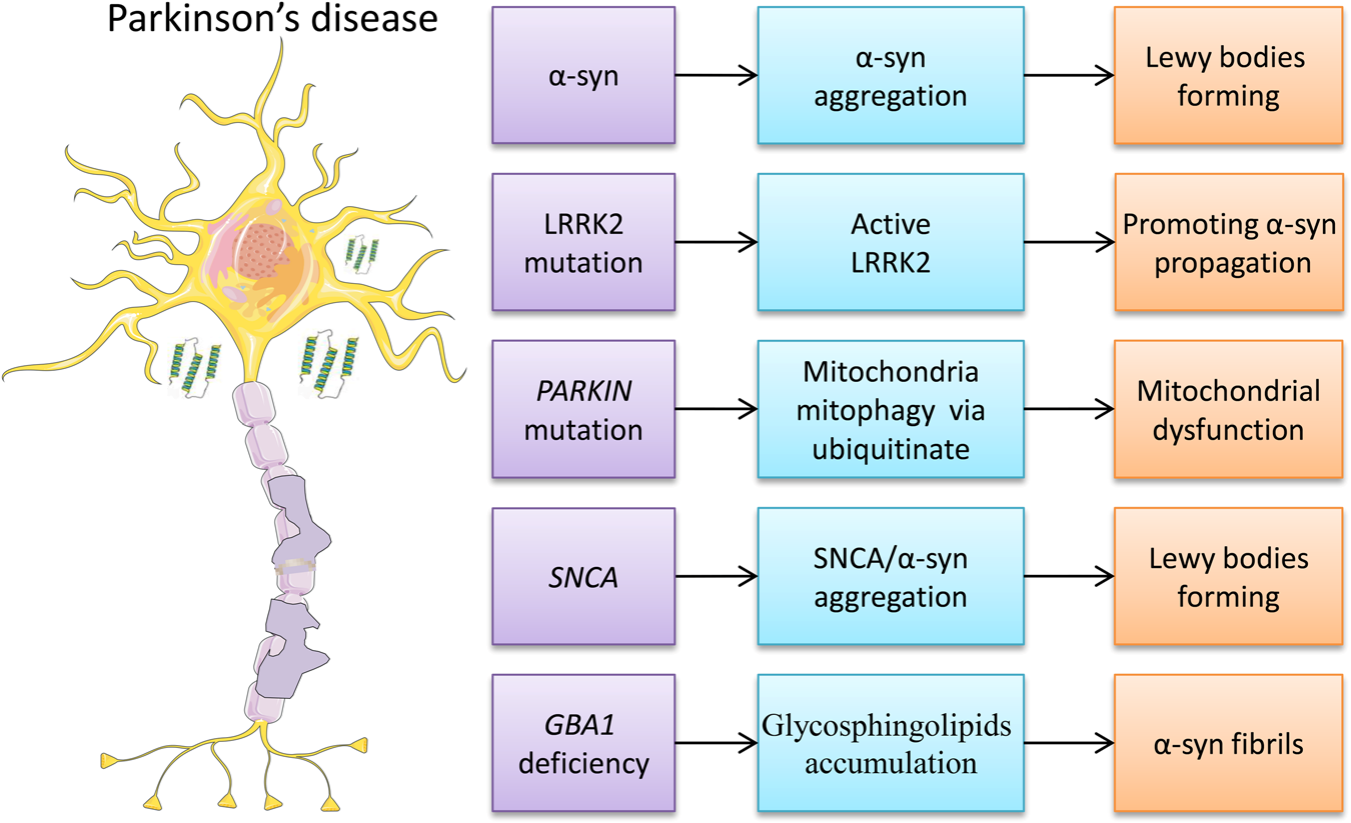
Associated markers or altered gene expression of PD. α-Synuclein, *LRRK2* mutation, *PARKIN* mutation, *SNCA*, and targeted deletion of *GBA1* have all been associated with PD (left column). The middle and right columns show the downstream effects.

Widespread aggregation of the α-synuclein protein in the form of Lewy bodies is a neuropathological hallmark of PD^28^ (Fig. 2). The coexistence of multiple pathological proteins in diseased brains is common in various neurodegenerative diseases, and one pathological protein could promote the spreading of another^29^. Approximately 50% of individuals with AD have α-synuclein, Aβ, and tau pathology in the brain^30^. Genetic and histopathological data suggest that Aβ plaques drive the spread of tau pathology^31^. Overexpression of α-synuclein has been observed to increase phosphorylated tau in mice^32^. Misfolded protein aggregates further activate the innate immune system in various neurodegenerative diseases. This implies that the inflammation induced by faulty protein clearance may be a common phenomenon in neurodegeneration. Astrocytes are activated by microglia via nuclear factor (NF)-κB signaling, thus further amplifying inflammation^33^, and exacerbating tissue damage and cellular dysfunction. High expression levels of tumor necrosis factor (TNF)-α and interleukin (IL)-1β have been linked to synaptic plasticity, learning, and memory^34^. Brain structure and function deteriorate via a feedforward loop that fuels further neurodegeneration, and no neuroprotective or neurorestorative therapies have been identified to date for treating neurodegenerative diseases. In this review, we discuss genetic and environmental risk factors for neurodegenerative diseases, summarize the communication among the components of the microbiota-gut-brain axis, and discuss the fecal microbiota transplantation (FMT) treatment strategy.

## Genetic and environmental risk factors for neurodegenerative diseases

Some genetic risk factors that modulate the transmission of pathological proteins contribute to the promotion of neurodegenerative disease development and progression. Targeted deletions of *CX3CR1* and *TREM2* variants^35^ and altered complement expression have been associated with neurodegenerative phenotypes^36^. CX3CR1 is the receptor for the chemokine fractalkine (CX3CL1) and is part of a critical signaling pathway for microglia-neuron crosstalk. The CX3CL1/CX3CR1 axis is implicated in the regulation of cognitive functions and synaptic plasticity in the hippocampus. CX3CR1 deficiency exacerbates α-synuclein-A53T-induced neuroinflammation and neurodegeneration in PD^37^. TREM2 is a transmembrane glycoprotein. TREM2 regulates phagocytic pathways and suppresses inflammatory reactivity to regulate the reactive microglial phenotype^38^. *TREM2* mutations decrease phagocytic activity, contribute to neurodegeneration by impairing the clearance of damaged neurons and aggregated proteins, and promote pro-inflammatory reactions^35^. It is estimated that the risk of AD attributable to genetic factors is 56–79%^39^. The role of epigenetic factors, DNA and histone modifications, and noncoding RNA in the development of neurodegenerative disease has been deeply investigated^40^. The *APOE* gene encoding apolipoprotein (Apo) E is the strongest genetic risk factor for developing AD. There are three predominant variants of *APOE*: *APOE2, APOE3,* and *APOE4*. People who carry the *APOE4* gene variant are at higher-than-average risk of developing AD, and the variant is linked to defects in the blood–brain barrier (BBB) and subsequent cognitive decline^41^. *APOE4* is associated with a fourfold increase in the risk of developing AD in people with one copy of this variant and a 15-fold increase in the risk of developing AD in people who have two copies^42^. Aβ and tau propagation are associated with ApoE^43^. The *BIN1* gene encoding amphiphysin 2, which can inhibit tau propagation, is the second most prevalent risk locus for late-onset AD^44^. Rare coding variants in *PLCG2*, *ABCA7*, *TREM2*, and *ABI3* have been identified in AD using genome-wide association studies (GWASs)^45^. The rare coding variants p.R62H (rs143332484) and p.R47H (rs75932628) in *TREM2* and p.P522R in *PLCG2* (rs72824905) are associated with the risk of AD^45,46^. Dysfunction in *TREM2* increases amyloid plaque seeding^47^. CX3CR1^+^ mononuclear phagocytes express antifungal receptors and activate antifungal responses in a Syk-dependent manner and are essential for mediating interactions between intestinal mycobiota and host immunity at steady-state and during inflammatory disease^48^ (Fig. 1). The CX3CR1/CX3CL1 axis plays a key role in the phagocytosis of tau by microglia and is affected as AD progresses^49^. In hTau/CX3CR1^−/−^ mouse models, microglial activation led to the secretion of IL-1, which promotes p38 MAPK-mediated tau hyperphosphorylation and aggregation^50^ (Fig. 1). A missense mutation in the gene encoding CX3CR1 led to changes in the gut fungal communities and to severe colitis, and impaired antifungal responses in Crohn’s disease patients^51^. G protein-coupled receptor 31 (GPR31) is highly and selectively expressed in intestinal CX3CR1^+^ cells. The bacterial metabolites lactic acid and pyruvic acid contribute to enhanced immune responses by inducing GPR31-mediated dendrite protrusion of intestinal CX3CR1^+^ cells^48^. Histone deacetylase 1 (HDAC1)-deficient mice display age-associated DNA damage accumulation and cognitive impairment. HDAC1 activation has the therapeutic potential for functional decline in brain aging and neurodegeneration^52^. Some studies have also shown that pathogens can act as triggers to induce the accumulation of Aβ_1–42_ monomers, reactive gliosis, and pro-inflammatory response, and are involved in the development of sporadic AD^53^.

Mutations in the gene encoding leucine-rich repeat kinase 2 (LRRK2) are the most common cause of hereditary PD^54^. LRRK2 activity promotes α-synuclein propagation via the phosphorylation of RAB35^55^. A genetic component for the apparently sporadic disease was not obvious in the early days of PD research. LRRK2 mutations can cause familial PD with age-dependent but variable penetrance; variants of the gene are also risk factors for sporadic PD^56^. Individuals with mutations in the genes *PARKIN*, *PINK1*, *SNCA*, *GBA1*, and *LRRK2* show an increased risk of developing familial PD^57^ (Fig. 2). In addition, multiple mutations in genes such as *C9orf72*, *TARDBP*, and *SOD1* are mainly expressed in a variety of nonneuronal cells which enhance immune dysregulation and neuroinflammation in the pathogenesis of ALS^58^.

Although considerable genetic research has highlighted the importance of copy number variations and de novo mutations in neurodegenerative disease etiology, environmental exposure has also been linked to the pathogenesis of these diseases^59^. Various environmental factors may modify and trigger psychiatric conditions. The burden of disease caused by environmental pollution is becoming a public health challenge worldwide, and 6.4 million deaths in 2015 were attributable to air pollution according to the Global Burden of Disease Study^60^. Experimental and epidemiologic evidence strongly supports the role of environmental exposure and gene–environment interactions in the incidence and progression of PD^61^. The toxicants of heavy metals, pesticides, detergents, solvents, and other industrial byproducts are highly relevant to neurologic disorders^62^. These toxicants can cross the BBB, potentially impacting the health and function of central nervous system (CNS) cells.

**Fig. 3:**
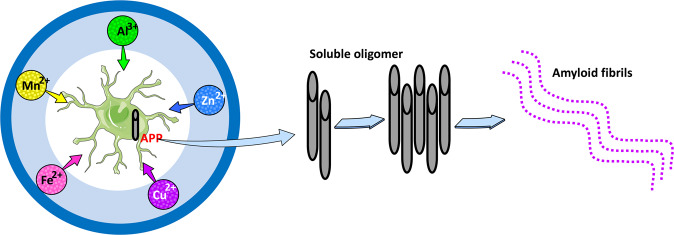
Implications of metals in Aβ neurotoxicity. APP binds to metals (Al, Cu, Fe, Mn, or Zn). Under acceleratory conditions, Aβ self-aggregates and forms several types of oligomers (SDS-soluble oligomers, Aβ-derived diffusible ligands, globulomers, and protofibrils) finally forming insoluble aggregates (amyloid fibrils), and tight binding to the surface of neurons and form fibrillar deposits.

Among all types of pollution, heavy metals are considered the greatest threat to human health because of their persistence and bioaccessibility in the environment. Increased industrialization has led to higher levels of heavy metals. Chronic exposure to transition metals such as manganese (Mn), iron (Fe), copper (Cu), and zinc (Zn) is linked to neurodegenerative disorders^63^. Conformational changes in disease-related proteins (Aβ, tau, and α-synuclein) are central to the pathogenesis of neurodegenerative diseases. The conformational changes of Aβ and its oligomerization are critical to the Aβ-induced neurodegeneration process. Aβ oligomers form insoluble aggregates termed amyloid fibrils. The pathogenesis of AD may be essentially altered by factors that accelerate oligomerization. Trace elements of Al^3+^, Zn^2+^, Cu^2+^, Mn^2+^, and Fe^2+^ are accelerating factors in protein conformational change; for example, they can enhance Aβ oligomerization^64^ (Fig. 3). Exposure to Mn^2+^ has been linked to an increased risk of neurodegenerative disorders according to our research and the research of others^65–67^. Mn^2+^ exposure promotes α-synuclein secretion and acts as a key amplifier of NLRP3 inflammasome signaling^63,68^. Mn^2+^ crosses the BBB as Mn^2+^ alone or in complex with transferrin or citrate^67^. Aluminum (Al) is a trivalent metal neurotoxin and is linked to the etiology of neurological disorders^69^. Al^3+^ enters the brain via a similar mechanism to Fe^2+^. Al^3+^ accumulation within the CNS induces pro-inflammatory signaling, irreversible brain cell damage, dysregulation of gene expression, and functional decline in cognition, memory, and behavior^70^. The mechanism of Al^3+^ toxicity is inflammatory neurodegeneration including amyloidogenesis, inflammasome activation, deficits in neurotrophic signaling and synaptogenesis, altered innate immune responses, reactive oxygen species (ROS), and α-synuclein production, and inability to remove self-aggregating waste from brain cells, cytoplasm, and parenchyma^71,72^. The imbalance of Zn^2+^ and Cu^2+^ plays a pivotal role in the mechanisms of AD and PD. Aβ aggregation and ROS production lead to excess intracellular Zn, release Zn from metallothioneins and may affect mitochondrial function and induce apoptosis. Excess Cu^2+^ is neurotoxic, and its neurotoxicity has traditionally been viewed as the result of its strong affinity for Aβ and its promotion of increased oxidative stress via the Fenton reaction^73^. Several studies have suggested that lead (Pb), arsenic (As), and methyl mercury (MeHg) are also neurotoxins and can disrupt brain function^74^, cause cognitive dysfunction^75^, and increase the risk of AD and PD by disrupting mRNA splicing, the ubiquitin-proteasome system, the electron transport chain, and oxidative stress^76,77^.

Pesticides are used to destroy, prevent, or control destructive pests. Food and Agriculture Organization (FAO) reported that ~3 million tons of pesticides are used globally every year. Exposure to pesticides has been identified as a risk factor for nervous system disorders, reproductive problems, and cancer linked to inflammasome activation^78^. Rotenone can easily cross the BBB and activate the NLRP3 inflammasome^79^, and rotenone exposure can cause nigrostriatal degeneration, α-synuclein accumulation, motor impairment, and neuroinflammation^80^. Paraquat is a widely used herbicide, and exposure to paraquat is also linked to an increased risk of PD and AD^62^. Paraquat induces ROS generation, cytotoxicity, and NLRP3 activation^81^. The organophosphate chlorpyrifos is widely used, and an estimated 3.2–4.1 million kilograms enter the environment annually in the United States alone^82^. Chlorpyrifos exposure can also increase the risk of PD by altering the expression of claudin5, ZO1, and TRPC4, which are important proteins for BBB integrity^83^.

The neurodegenerative process can be exacerbated by neuroinflammation^84^. Inflammasomes sense damage-associated molecular patterns and pathogen-associated molecular patterns. Growing evidence indicates that there is an association between inflammasome activation and neurodegenerative disease. Heavy metals and pesticides cause cellular damage by deregulating lysosomal function, impairing mitochondrial function, enhancing the spread of misfolded proteins, and potentially triggering an inflammatory response ranging from the induction of acute necrosis to more discrete cellular pathophysiologies, including protein misfolding, oxidative stress, and programmed cell death^85^. Inflammasomes may link environmental toxicant-driven cellular stress with neuroinflammation and ultimately cell death.

## Genetic and environmental factors affect gut microbiota

The gut microbiota is a dynamic microbial system, and it can be modified by genetic and environmental factors. Previous studies have reported that the gut microbiota is constantly challenged by environmental factors such as exercise, diet, stress, altitude, temperature, toxicants/pollutants, and noise^86,87^. Environmental contaminants (heavy metals, pesticides, persistent organic pollutants, antibiotics, food additives, and nanomaterials) can affect the composition of the gut microbiota, leading to physiological disorders in the host and causing certain diseases^88^. The gut microbiota has become a new toxicological target for some environmental pollutants. A decreasing diversity of gut microbiota is often observed after exposure to heavy metals^89^. In our previous studies, Mn exposure led to decreased abundances of *Prevotellaceae*, *Fusobacteriaceae*, and *Lactobacillaceae*^66,90^. In addition, Nasuti et al.^91^ showed that changes in gut microbiota may be one of the reasons for the neurotoxicity of permethrin. Many studies have shown that antibiotic administration leads to disturbances in the microbial diversity and metabolism of the gut microbiome that might be linked to a multitude of diseases^89^.

The host’s genetic background can influence microbiota composition. The microbiomes of humans and mice are associated with host genetic variation, and several heritable bacterial taxa have been identified^92,93^. The gut microbiota, as an epigenetic factor influencing DNA methylation status in the *SNCA* promoter region, may affect α-synuclein expression and the risk of PD^94^. The *APOE* genotype, by influencing bile acid secretion, could affect the composition of the gut microbiota to favor the development of organisms triggering protein misfolding, increasing the risk for PD in synucleinopathies^95,96^. Moreover, TAS2R38 has been shown to be a genetic risk factor associated with the development of PD. Genetic variants of the TAS2R38 bitter taste receptor are associated with distinct gut microbiota traits in PD and are associated with a reduction in bacterial alpha diversity with a predominant reduction in the *Clostridium* genus^97^. The relative abundance of certain microbiota elements can be influenced by the genetic background of the subject, as demonstrated in a large study of monozygotic and dizygotic twins^98^. The *APOE* genotype is the strongest prevalent genetic risk factor for AD. Structural and specific gut microbiome profiles were strongly and significantly associated with *APOE* alleles^99,100^. Tran et al.^99^ reported that different *APOE* genotypes can influence the relative abundances of several bacterial taxa, such as *Prevotellaceae* and *Ruminococcaceae* and several butyrate-producing genera, in both humans and transgenic mice. Guardia-Escote et al.^101^ also showed that the composition of gut microbiota in early life can be modulated by the *APOE* genetic background. Environmental factors such as dietary habits, living conditions, and contamination of environmental matrices can also interact with genetic profiles to affect gut microbiota composition^93,102^.

Diet is a principal environmental factor and an established modulator that influences gut microbiota composition^103^. Various dietary patterns, nutrients, and food components have the potential to substantially alter the gut microbiota composition. For example, the gut microbiota composition appears to be sensitive to caloric balance^104^. Cohousing mice harboring an obese twin’s microbiota with mice containing the lean co-twin’s microbiota fed low saturated fat, high fruit and vegetable diet can take on microbiota characteristics of lean mice^105^. High energy-dense diet rapidly altered the gut microbiota composition with increases in pro-inflammatory *Proteobacteria* proliferation and in *Firmicutes*/*Bacteroidetes* ratio in rats^106^. Moreover, a rapid shift in gut microbiota composition was observed in humans, with increased abundances of *Alistipes*, *Bilophila*, and *Bacteroides*, after consuming a high-fat/protein diet for 5 days^103^, and *Bacteroides spp*. are highly associated with animal proteins, but *Prevotella spp*. are highly associated with increased intakes of plant proteins^107^.

## The microbiota-gut-brain axis

The gut-brain axis is a network comprising the gastrointestinal tract, the enteric nervous system (ENS), and the brain. Immunity, digestion, metabolism, satiety, and stress reactions can be regulated by bidirectional communication along the gut-brain axis^108^. Gut bacteria have been found to play crucial roles in neurodevelopment, neuroinflammation, and behavior^109^. A growing body of research has focused on the microbiota-gut-brain axis. The vagus nerve synapses on enteric neurons and enables gut-brain communication. The dysregulation of the microbiota-gut-brain axis has been increasingly implicated in psychiatric and neurological disorders, such as AD^110^, PD^111^, stroke^109^, and multiple sclerosis^109^. Gut microbial products can affect neuronal transcription and thus host behavior via gene–environment interactions^112,113^. For example, γ-aminobutyric acid (GABA), tryptophan, serotonin, histamine, and dopamine, which are neurotransmitters or precursors in the brain, can directly affect how neurons communicate with each other. The microbiota-gut-brain axis is a potential new therapeutic target for the effective treatment of CNS disorders via the immune system, direct ENS routes, and the vagus nerve by altering the recruitment of host neurochemical signaling and the production of bacterial metabolites^108^. Microbial metabolites are often most markedly altered in the disease state, and such metabolites [e.g., short-chain fatty acids (SCFAs), tryptophan, tyrosine derivatives, and trimethylamine N-oxide] have significant effects on physiological processes^114^.

**Fig. 4:**
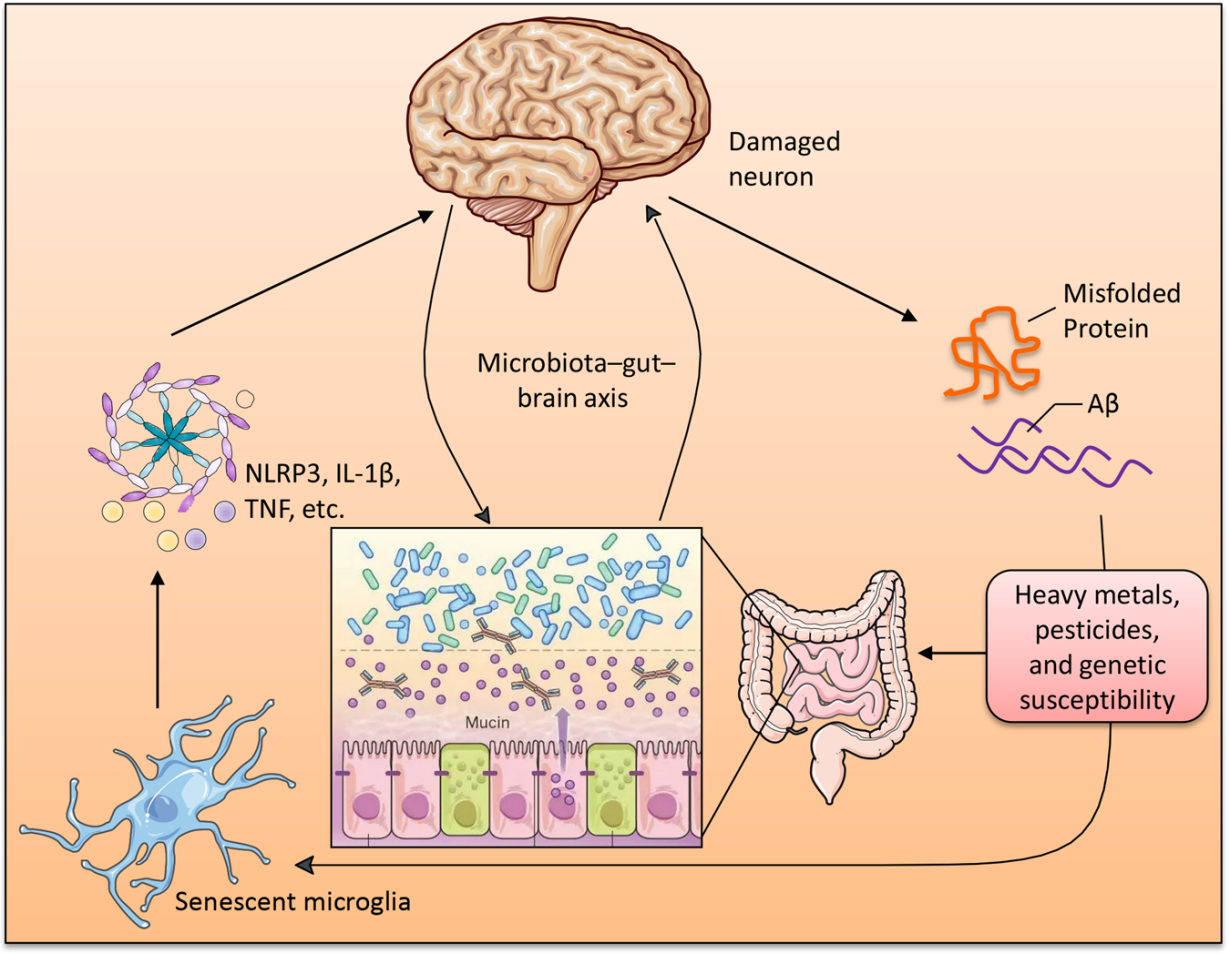
The microglia is a key mediator in the communication among microbiota-gut-brain axis. Damaged neurons in the neurodegenerative brain release Aβ, tau, or α-synuclein, which accumulate and activate microglia. This leads to activation of senescent microglia that produce increased levels of pro-inflammatory cytokines and accelerating inflammatory cascade.

There are many bidirectional communication pathways between the gut microbiota and the brain, including the autonomic nervous system (ANS), ENS, immune-modulatory responses, enteroendocrine signaling, neurotransmitters, and microbial metabolite signaling^115^. The ANS coordinates with the hypothalamic-pituitary-adrenal (HPA) axis to promote integrated communication between the brain and the gut, which is responsible for endocrine and physiological homeostasis and autonomic, motor, and behavioral functions. The ENS communicates with the CNS via intestinofugal neurons^116^. Enteroendocrine cells, such as enteroendocrine L cells and enterochromaffin cells, are essential for maintaining gut homeostasis; they can establish direct contact with luminal constituents via the apical surface^117^. Microbiota-derived neuromodulatory metabolites include catecholamines, histamine, 5-hydroxytryptamine (5-HT), GABA, and tryptophan precursors and metabolites, which are involved in host mood, behavior, and cognition^118^. Branched-chain amino acids (BCAAs) participate in a variety of biochemical functions in the peripheral and CNS^119,120^. BCAAs enhance protein synthesis through the mTOR signaling pathway, reduce protein oxidation, and have positive effects on mitochondrial biogenesis and ROS scavenging^120^. SCFAs are key players in microbiota-gut-brain axis communication that influence intestinal mucosal immunity, barrier integrity, and function, as well as BBB integrity and neuroinflammation^121^. Acetate, propionate, and butyrate are the most abundant SCFAs in the human body, and SCFAs might influence the microbiota-gut-brain axis via interaction with free fatty acid receptors (FFARs) and/or inhibition of histone deacetylases (HDACs)^122^. SCFAs have been implicated in gastrointestinal function^123^, immune function^124^, autism spectrum disorder (ASD)^122^, PD^125^, and AD^126^. SCFAs interact with gut mucosal enteroendocrine cells and can migrate into the CNS^127^. Neuromodulators, SCFAs, bile acids, bacteriocins, and choline are immunomodulatory and activate the innate immune system. Pro-inflammatory cytokines within the brain are released when the innate immune response is activated^128^. In turn, astrocytes are activated by microglia via NF-κB signaling due to the upregulation of these pro-inflammatory cytokines^30^, leading to further amplification of inflammation and the immune response^129^, deterioration of brain structure and function, and disease pathology^130^ (Fig. 4). A recent study found that Aβ deposits were observed in the gastrointestinal tract of AD patients and transgenic mice overexpressing APP^131,132^. Enteric Αβ directly induces cerebral amyloidosis and AD-like dementia may be by retrograde axonal transportation via the vagus^132^.

## Fecal microbiota transplantation in neurodegenerative diseases

Research on the role of the gut microbiome in regulating brain function is growing rapidly. The microbiome may be a key susceptibility factor for neurodegenerative diseases. Perturbations of the gut microbiota are associated with multiple diseases. The microbiota in the gastrointestinal tract impacts the development and function of the nervous, metabolic, and immune systems^133^. Microbiome reconfiguration can alter its function and may modify disease symptoms^134^. Transplantation of microbiota from patients with ASD^135^, schizophrenia (SCZ)^136^, and irritable bowel syndrome (IBS) into wild-type mice promoted indication-specific behavioral symptoms^137^, such as hallmark autistic behaviors for ASD; locomotor hyperactivity decreased anxiety and depressive-like behaviors, and increased startle responses for SCZ; and faster gastrointestinal transit, intestinal barrier dysfunction, innate immune activation, and anxiety-like behavior for IBS. FMT and antibiotic and probiotic interventions have shown promise for the treatment of neurodegenerative diseases in limited human trials. FMT is a procedure in which stool from a healthy donor is placed into another patient’s intestine^138^. FMT from a healthy donor resolved recurrent *Clostridioides difficile* infections and was suggested to prevent multiple sclerosis disease progression for over 10 years^139^. FMT temporarily improved leg tremors and other PD symptoms in a PD patient^140^. Xue et al.^141^ reported that FMT via colonoscopy can relieve the motor and non-motor symptoms with acceptable safety in PD patients based on a small-scale trial. FMT cured epilepsy in a case with Crohn’s disease^142^. Many animal studies have suggested a positive effect of FMT on neurodegenerative diseases.

Germ-free wild-type mice and their offspring had ASD-like symptoms and displayed alternative splicing of ASD-relevant genes when FMT was performed with stool from children with ASD. ASD symptoms decreased when GABA receptor agonists were administered to the ASD model^135^. Decreased cerebral oxidative stress was observed in another study after FMT from a normal hamster in an ASD hamster model^143^.

Gut bacteria control the differentiation and function of immune cells^144,145^. Braak’s hypothesis posits that PD may start in the gut, triggered by a pathogen, and spread to the brain^146^. Gut microbiota dysbiosis is linked to PD^134^. Sampson et al.^134^ reported that gut microbiota are required for motor deficits, microglial activation, and α-synuclein pathology in an α-synuclein-overexpressing mouse model, revealing that alterations in the human microbiome represent a risk factor for PD. Elevated levels of probiotics and depletion of anti-inflammatory SCFA-producing bacteria have been confirmed in PD patients^147^. α-Synuclein-overexpressing germ-free mice that underwent FMT with stool from PD-affected patients exhibited enhanced physical impairments^134^. The PD mice model showed improved motor function in the pole descent test and traction test and inhibited the TLR4/TBK1/NF-κB/TNF-α signaling pathway-mediated gut inflammation and neuroinflammation after receiving feces from healthy mice in another study^148^. Conversely, wild-type mice administered fecal matter from PD mice displayed impaired motor function and decreased striatal dopamine and serotonin levels, while FMT had no side effects on behavioral functions and neurotransmitters in normal mice^148^.

Another study found that the seizure threshold increased after transplantation with *Parabacteroides merdae*, *Akkermansia muciniphila*, and *Parabacteroides distasonis* in mice^149^. There are numerous publications about the relationship between AD and gut microbiota. The composition of the gut microbiota of AD patients differed from that of healthy controls at the taxonomic level, such as *Bacteroides*, *Actinobacteria*, *Ruminococcus*, *Lachnospiraceae*, and *Selenomonadales*^150^. Gut microbial alterations have been associated with cognitive impairment^151^ and Aβ load^110^ in older adults. Gut microbiota alterations may stimulate inflammatory pathways that trigger neuroinflammation^152^. The pro-inflammatory cytokines IL-6, CXCL2, NLRP3, and IL-1β and the anti-inflammatory cytokine IL-10 are released by TLRs, and they can cross the BBB via both diffusion and cytokine transporters^110^. Patients with cognitive impairment and brain amyloidosis have more pro-inflammatory gut bacteria in their feces^110^. Furthermore, germ-free wild-type mice that received AD feces showed lower levels of neuro-related fecal metabolites and poorer cognitive function^153^. Microbial-mediated intestinal and systemic immune dysfunction is an important component of the pathogenesis of AD, and FMT from healthy wild-type mice into transgenic AD model mice with AD-like pathology, amyloid deposits, and neurofibrillary tangles alleviated the formation of Aβ plaques and neurofibrillary tangles, glial reactivity, and cognitive impairment^154^.

FMT may reverse the decrease in cognitive function induced by antibiotics. Wild-type mice showed a cognitive decline after broad-spectrum antibiotic therapy. However, memory and spatial learning were improved after receiving anti-aging mouse feces^155^. Human microbiome transplantation protected germ-free mice from death caused by acute arsenic toxicity^156^. According to our research, Mn exposure increased Aβ and inflammatory factor production in the brain and caused hippocampal degeneration and necrosis^66,90^. FMT from normal rats alleviated the neurotoxic effects of Mn exposure by altering the gut microbiota^66^.

The literature suggests a potential beneficial effect of healthy donor FMT. FMT may be a promising treatment option for neurodegenerative diseases, and restoration of the gut microbiota to a premorbid state is a novel goal for prevention and treatment strategies^157^. However, for microbiome-linked diseases, the gut microbiota required for successful treatment remains unknown. When preparing FMT, careful measures should be taken to maintain the viability of the diverse bacterial population. Meanwhile, inherent risks of FMT include the possibility of aspiration with bowel perforation after a colonoscopy and upper gastrointestinal delivery^158^. Some mild gastrointestinal symptoms have been reported after FMT, including constipation, diarrhea, fever, abdominal discomfort, flatulence, bloating, belching, vomiting, nausea, and borborygmus^159^. FMT also has the risk of infection transmission^160^, such as bacterial translocation, and bacterial infections caused by multidrug-resistant organisms. With the COVID-19 pandemic, FMT could potentially transmit SARS-CoV-2. SARS-CoV-2 genetic material, including live virus, can be detected in feces even after the resolution of respiratory symptoms^161,162^. Autoimmune diseases and metabolic syndrome are associated with disturbances in the gut microbiome and should also be assessed as potential long-term risks related to FMT^158^.

## Conclusions

Despite significant advances in our understanding of the pathobiology of neurodegenerative diseases, pathobiology is complex, and we have not yet identified an effective treatment for neurodegenerative diseases in humans. It has been revealed that bidirectional communication exists between the brain and the gut. The microbiota in the gastrointestinal tract impacts the development and functions of the immune, metabolic, and nervous systems and is associated with multiple diseases. The latest findings reviewed here improve our understanding of the genetic and environmental risk factors of neurodegenerative disease. Moreover, the discovery of the communication among the components of the microbiota-gut-brain axis has led to the idea of ameliorating neurodegenerative diseases by FMT. FMT may be a promising treatment option with great potential to treat neurodegenerative diseases in the future. However, we should also be aware that FMT could increase the risk of bacterial translocation, sepsis, and bacterial infections caused by multidrug-resistant organisms.

### Reporting Summary

Further information on research design is available in the Nature Research Reporting Summary linked to this article.

## Supplementary information

Reporting Summary
